# Structure-Function Relationship for a Divergent Atg8 Protein Required for a Nonautophagic Function in Apicomplexan Parasites

**DOI:** 10.1128/mbio.03642-21

**Published:** 2023-01-10

**Authors:** Marta Walczak, Thomas R. Meister, Hoa Mai Nguyen, Yili Zhu, Sébastien Besteiro, Ellen Yeh

**Affiliations:** a Department of Pathology, Stanford School of Medicine, Stanford, California, USA; b Department of Molecular and Cellular Physiology, Stanford School of Medicine, Stanford, California, USA; c LPHI UMR5235, University of Montpellier, CNRS, Montpellier, France; d Department of Microbiology & Immunology, Stanford School of Medicine, Stanford, California, USA; e Chan Zuckerberg Biohub, San Francisco, California, USA; University of Pittsburgh

**Keywords:** Atg8, *Plasmodium*, *Toxoplasma*, apicomplexan parasites, apicoplast, malaria, nonautophagic function of Atg8

## Abstract

Atg8 family proteins are highly conserved eukaryotic proteins with diverse autophagy and nonautophagic functions in eukaryotes. While the structural features required for conserved autophagy functions of Atg8 are well established, little is known about the molecular changes that facilitated acquisition of divergent, nonautophagic functions of Atg8. The malaria parasite Plasmodium falciparum offers a unique opportunity to study nonautophagic functions of Atg8 family proteins because it encodes a single Atg8 homolog whose only essential function is in the inheritance of an unusual secondary plastid called the apicoplast. Here, we used functional complementation to investigate the structure-function relationship for this divergent Atg8 protein. We showed that the LC3-interacting region (LIR) docking site (LDS), the major interaction interface of the Atg8 protein family, is required for P. falciparum Atg8 (*Pf*Atg8) apicoplast localization and function, likely via Atg8 lipidation. On the other hand, another region previously implicated in canonical Atg8 interactions, the N-terminal helix, is not required for apicoplast-specific *Pf*Atg8 function. Finally, our investigations at the cellular level demonstrate that the unique apicomplexan-specific loop, previously implicated in interaction with membrane conjugation machinery in recombinant protein-based *in vitro* assays, is not required for membrane conjugation nor for the apicoplast-specific effector function of Atg8 in both P. falciparum and related Apicomplexa member Toxoplasma gondii. These results suggest that the effector function of apicomplexan Atg8 is mediated by structural features distinct from those previously identified for macroautophagy and selective autophagy functions.

## INTRODUCTION

Atg8 family proteins are highly conserved eukaryotic proteins with important functions in autophagy and nonautophagic pathways in eukaryotes (1, 2). This small, ubiquitin-like protein is covalently conjugated to membrane lipids where it recruits effectors, lying at the nexus of protein interactions to coordinate membrane dynamics (1, 3, 4). Although their role in autophagy is the best defined, Atg8 family proteins are functionally diverse. Atg8 and its conjugation machinery (Atg4, Atg3, and Atg7) are ubiquitous among eukaryotes, suggesting that autophagy or another Atg8-associated membrane function was a core pathway present in the last common eukaryotic ancestor (5, 6). From this highly conserved starting point, in contrast to the single ortholog found in fungi and some protists, the Atg8 family has expanded to multiple paralogs in mammals and plants, hypothesized to represent functional specialization and diversification (7, 8).

In humans at least six Atg8 homologs have been identified: LC3A, LC3B, LC3C, GABARAP, GABARAPL1/GEC1, and GABARAPL2/GATE-16 play partly redundant roles in autophagy but also participate in a number of nonautophagic pathways such as phagocytosis, vesicle trafficking, secretion, and exocytosis that do not overlap across homologs (2, 3, 9). These nonautophagic functions highlight the integral role of the Atg8 protein family in the mammalian endomembrane system beyond their role in autophagy. Unfortunately, overlapping autophagy and nonautophagic functions of mammalian Atg8 homologs make it challenging to investigate the functional diversification of this conserved protein family.

Atg8 proteins have also evolved a nonautophagic function in a distinct endomembrane compartment in a group of divergent eukaryotes. *Plasmodium* spp., the protists that cause malaria, and related apicomplexan parasites such as Toxoplasma gondii contain a nonphotosynthetic plastid derived from secondary endosymbiosis called the apicoplast. During secondary endosymbiosis, a chloroplast-containing alga was phagocytosed by another eukaryote and established as a multimembrane plastid that is part of the endomembrane system (10, 11). Unexpectedly, the single Atg8 homolog in T. gondii was shown to be conjugated to the outer membrane of the apicoplast (12). Although Atg8-dependent autophagy-like pathways have been suggested in blood-stage Plasmodium falciparum and intrahepatocytic Plasmodium berghei (13, 14), it is unclear if canonical macroautophagy occurs in *Plasmodium* since core components of the initiation steps and typical lysosomes are missing (15). Instead, knockdown of Atg8 in blood-stage P. falciparum causes loss of the apicoplast during cell division and parasite death. When apicoplast function is rescued in Atg8-deficient parasites, parasite growth is recovered, demonstrating that the novel function of P. falciparum Atg8 (*Pf*Atg8) in apicoplast inheritance is its only essential function, at least in the blood stage of the parasite life cycle (16).

*Pf*Atg8’s novel apicoplast function offers a unique opportunity to investigate Atg8 protein structure-function relationships for a nonautophagic function in the absence of autophagy. Atg8 homologs are compact proteins of <150 amino acids. Previous protein structure-function studies have defined several Atg8 protein regions important for autophagy (see Fig. S1 in the supplemental material): (i) an Atg8-interacting motif/LC3-interacting region (LIR) docking site (LDS) that is a key binding pocket for cargo receptors and core autophagy machinery; (ii) an N-terminal helical extension, a hallmark of Atg8 proteins distinguishing them from other members of the ubiquitin superfamily, important for oligomerization, membrane tethering, and fusion; and most recently (iii) a ubiquitin-interacting motif (UIM) docking site (UDS), which interacts with several new autophagy receptors (17–20). These structure-function relationships have provided important molecular insight into functional specialization for selective autophagy, for example, mutation of the LDS to modulate affinity and selectivity for specific cargo receptors (21, 22).

10.1128/mbio.03642-21.1 FIG S1 (A) Clustal alignment of P. falciparum (*Pf*), human (*Hs*), and yeast (*Sc*) Atg8 homologs. The additional amino acids in the apicomplexan-specific loop of *Pf*Atg8 are in green; amino acids corresponding to the loop in canonical homologs are in purple; residues of the *Pf*Atg8 LDS are in red; N-terminal helix α1 and the UDS are highlighted with cyan boxes; amino acids HQH are in orange boxes. The inset shows *Pf*Atg8 crystal structure (4EOY) with features color coded as in the alignment. (B) Clustal alignment of P. falciparum (*Pf*), T. gondii (*Tg*), and C. parvum (*Cp*) Atg8 homologs. The apicomplexan-specific loop is highlighted in bold red. Amino acids corresponding to the N-terminal helix α1, the UDS, and HQH are highlighted. For accession numbers of the aligned sequences, see “Cloning of *Plasmodium* constructs” in Materials and Methods. Download FIG S1, EPS file, 2.6 MB. Copyright © 2023 Walczak et al. 2023 Walczak et al. https://creativecommons.org/licenses/by/4.0/ This content is distributed under the terms of the Creative Commons Attribution 4.0 International license.

A significant gap in our knowledge is the molecular determinants important for nonautophagic functions. Do effectors also engage known LDSs or UDSs for nonautophagic functions? Are membrane tethering and fusion by the N-terminal extension selective for certain endomembrane compartments? More broadly, how have nonautophagic functions contributed to functional diversification and expansion of Atg8 protein families? By deciphering the molecular basis of *Pf*Atg8’s nonautophagic function at the apicoplast, we sought to provide insights into the molecular evolution that facilitated functional diversification in this important protein family.

## RESULTS

### Atg8 homologs with specialized autophagic and nonautophagic functions cannot complement the apicoplast function of *Pf*Atg8.

Atg8 homologs from distantly related species often can functionally complement autophagic functions in yeast and/or mammalian cells. Atg8 homologs from Leishmania major, Trypanosoma cruzi, and Arabidopsis thaliana have been able to at least partly restore autophagic function in *atg8*Δ yeast (23–26). These functional complementation experiments suggest that the structural features required for autophagic function are highly conserved among Atg8 homologs.

*Pf*Atg8 shares 33 to 43% sequence identity and 50 to 80% similarity with yeast and mammalian Atg8 homologs (see Fig. S1 in the supplemental material). While the function of yeast Atg8 is limited to autophagy pathways, mammalian homologs show functional diversification. This is likely due to structural differences which affect the selectivity and specificity of their interactions in both autophagic and nonautophagic functions. We wondered whether mammalian Atg8 homologs that showed functional diversification in autophagy and nonautophagic pathways could complement *Pf*Atg8’s function in the apicoplast. Of note, *Pf*Atg8 could partially complement *atg8*Δ yeast, while its P. berghei counterpart failed to do so (23, 27, 28).

**FIG 1:**
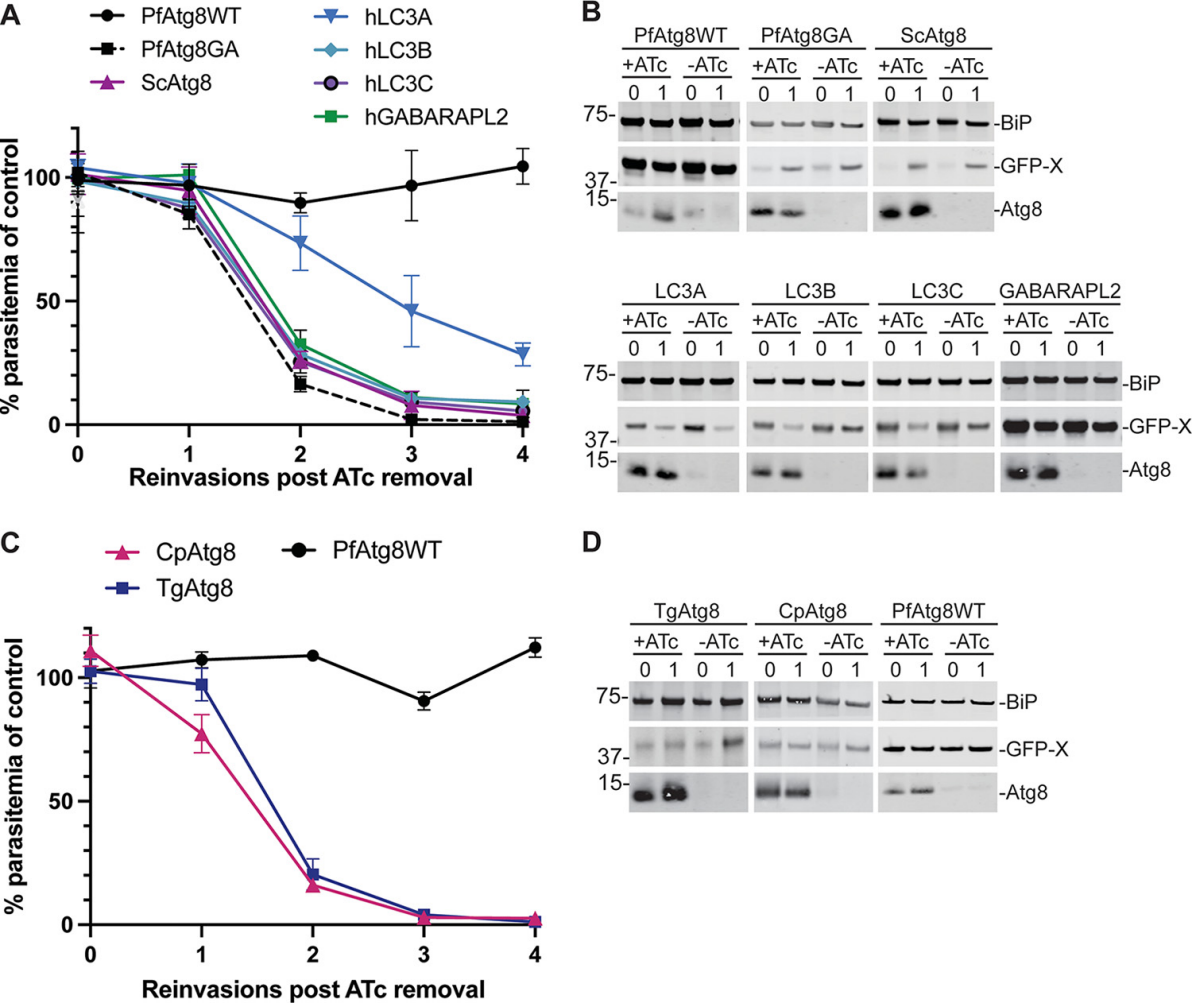
Atg8 homologs from other species do not efficiently complement *Pf*Atg8 function. (A) Growth of Atg8-TetR/DOZI parasites expressing indicated GFP-tagged homologs from human and yeast Saccharomyces cerevisiae (h and Sc, respectively) in the absence of ATc over 4 reinvasion cycles. Parasitemia for each time point was normalized to the culture grown in the presence of ATc. Mean ± standard deviation from a minimum of 2 biological replicates is shown. (B) Western blots showing expression of indicated proteins during the first two cycles of the growth time course. Equal parasite numbers were loaded per lane. BiP, loading control; X, Atg8 homolog. Numbers above the blots represent parasite reinvasion cycles after ATc removal. Numbers beside blots here and throughout figures indicate molecular masses in kilodaltons. (C) Growth of Atg8-TetR/DOZI parasites expressing GFP-tagged *Tg*Atg8, *Cp*Atg8, or *Pf*Atg8 in the absence of ATc over 4 reinvasion cycles. Parasitemia for each time point was normalized to the culture grown in the presence of ATc. Mean ± standard deviation for 2 biological replicates is shown. (D) Western blots showing Atg8 knockdown and expression of indicated GFP-tagged proteins during the first two cycles of the growth time course. Equal parasite numbers were loaded per lane. BiP, loading control. Numbers above the blots represent parasite reinvasion cycles after ATc removal.

Episomes expressing Atg8 homologs from these different species were introduced into a *Pf*Atg8-TetR/DOZI strain, in which *Pf*Atg8 expression is regulated by anhydrotetracycline (ATc) via the TetR/DOZI repressor (16, 29). Each transgenic Atg8 homolog was expressed as an N-terminal green fluorescent protein (GFP) fusion protein and was truncated to remove the final amino acids in the native sequence, exposing the terminal Gly residue that is conjugated to lipids (4). These constructs mimic the membrane conjugation of *Pf*Atg8, whose native sequence ends in a terminal Gly residue and does not require processing by the cysteine protease *Pf*Atg4 prior to membrane conjugation (16, 30). Successful transfectants were obtained for episomes expressing *Pf*Atg8WT (positive control), *Pf*Atg8G124A mutant (negative control which cannot be conjugated to the membrane), Saccharomyces cerevisiae Atg8 (*Sc*Atg8), and human LC3A, LC3B, LC3C, and GABARAPL2/GATE-16. While transgenic GFP-*Pf*Atg8WT was able to fully restore parasites’ growth upon loss of *Pf*Atg8 expression, neither yeast Atg8 nor human LC3B, LC3C, or GABARAP-L2 was able to restore growth in the absence of endogenous *Pf*Atg8 (Fig. 1A and B). Parasites expressing GFP-LC3A showed a weak rescue phenotype with 28% growth compared to control parasites (grown in the presence of endogenous Atg8) after 4 reinvasion cycles.

T. gondii and Cryptosporidium parvum are apicomplexan parasites related to *Plasmodium* spp. T. gondii also has an apicoplast and T. gondii Atg8 (*Tg*Atg8) is required for its inheritance, suggesting a similar nonautophagic function for *Pf*Atg8 and *Tg*Atg8 (12). C. parvum, on the other hand, lost the apicoplast in the course of evolution, although it retained a copy of the Atg8 gene (10). Both *Tg*Atg8 and C. parvum Atg8 (*Cp*Atg8) have >60% sequence identity to *Pf*Atg8 (Fig. S1). We were interested if the Atg8 homologs from either of these two closely related parasites can functionally complement *Plasmodium* Atg8. To this end, episomes containing GFP-*Tg*Atg8 or GFP-*Cp*Atg8 terminated with a Gly residue were transfected into the *Pf*Atg8-TetR/DOZI strain. Surprisingly, neither of the homologs could restore growth in *Plasmodium* lacking endogenous Atg8 (Fig. 1C and D). Furthermore, only one out of three transfections with GFP-*Tg*Atg8 was successful, suggesting that the construct may be toxic to *Plasmodium*.

Altogether, these data suggest that *Plasmodium* Atg8 has structural features absent from other, even closely related, homologs that are specifically required for its function in the *Plasmodium* apicoplast. The lack of efficient complementation of *Pf*Atg8 function by yeast, mammalian, and apicomplexan homologs could be due to protein expression, misfolding, or degradation, or loss of membrane conjugation or, once attached to the membrane, inability to interact with effectors.

### Atg8 conjugation to the apicoplast membrane is specific but does not depend on the apicomplexan-specific loop.

**FIG 2:**
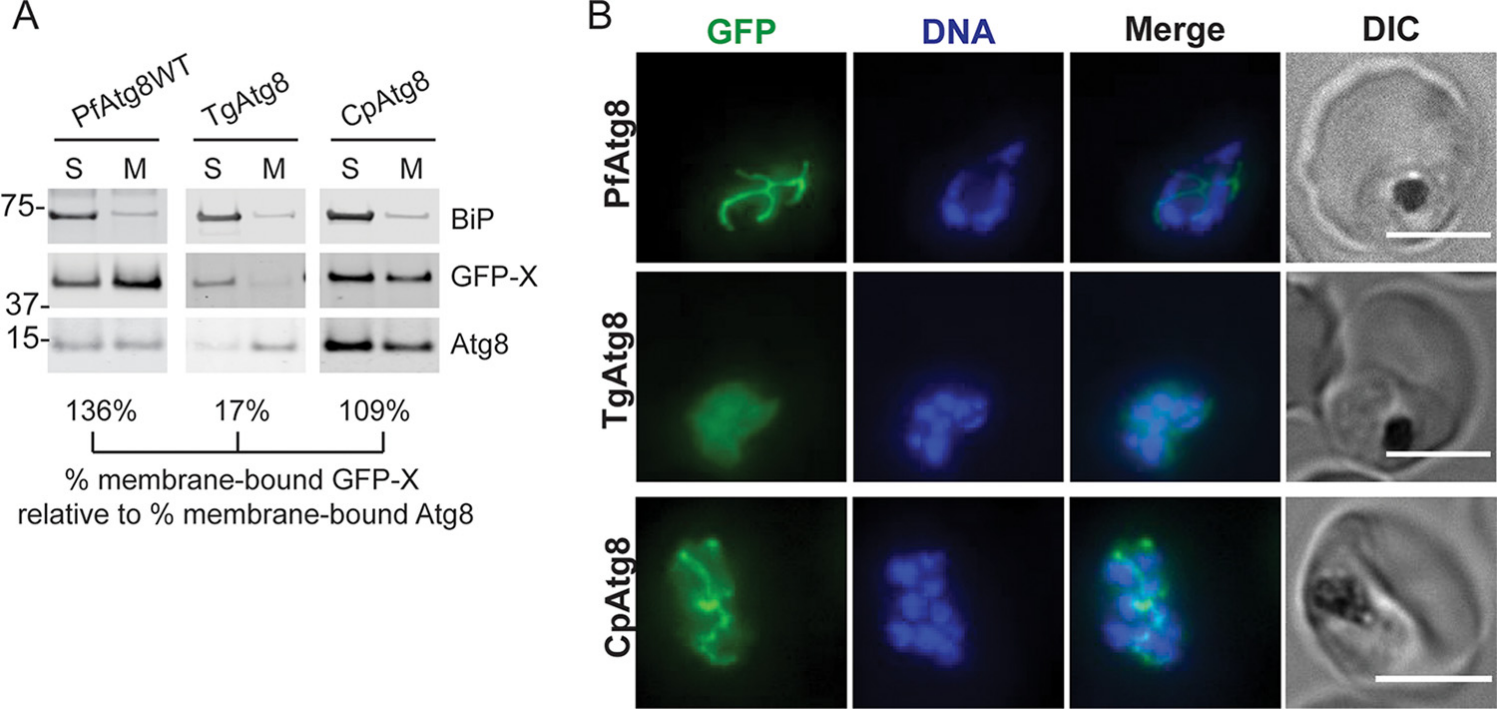
*Cp*Atg8, which does not functionally complement *Pf*Atg8 function, correctly localizes to the apicoplast membrane. (A) Western blot showing membrane association of selected proteins in a Triton X-114 fractionation experiment. Numbers below the blots represent the percentage of membrane-bound GFP-tagged Atg8 homolog normalized to percentage of the membrane-bound endogenous Atg8. S, soluble fraction; M, membrane fraction; BiP, soluble protein; Atg8, membrane-bound protein; X, Atg8 homolog. (B) Representative live fluorescence images showing the localization of selected GFP-tagged *Pf*Atg8 homologs. DNA was stained with Hoechst 33342. Maximum-intensity projections are shown. Bar, 5 μm. DIC, differential inference contrast.

The apicoplast function of *Pf*Atg8 requires its recognition and attachment to the apicoplast membrane via the action of membrane conjugation machinery, Atg3, Atg7, and a noncovalent Atg12-Atg5 complex (31, 32). Since none of the Atg8 homologs we tested could fully complement *Pf*Atg8, we screened these homologs for membrane association and apicoplast localization. To determine membrane association, cells expressing GFP-Atg8 fusions were subjected to Triton X-114 fractionation. This assay utilizes temperature-dependent phase separation of Triton X-114 into a detergent-rich fraction containing membrane-bound proteins and an aqueous fraction containing soluble and peripheral membrane proteins which are then analyzed by Western blotting (33). Because partitioning of proteins between the two fractions was variable between experiments, we normalized the percentage of membrane-bound GFP-Atg8 homolog fusions to the percentage of membrane-bound endogenous Atg8. To determine apicoplast localization, GFP-tagged Atg8 homologs were analyzed by live microscopy. Of all tested homologs, only *Cp*Atg8 showed membrane association and a branched localization reminiscent of the apicoplast in late schizont stages (34), similar to *Pf*Atg8 (Fig. 2A and B).

**FIG 3:**
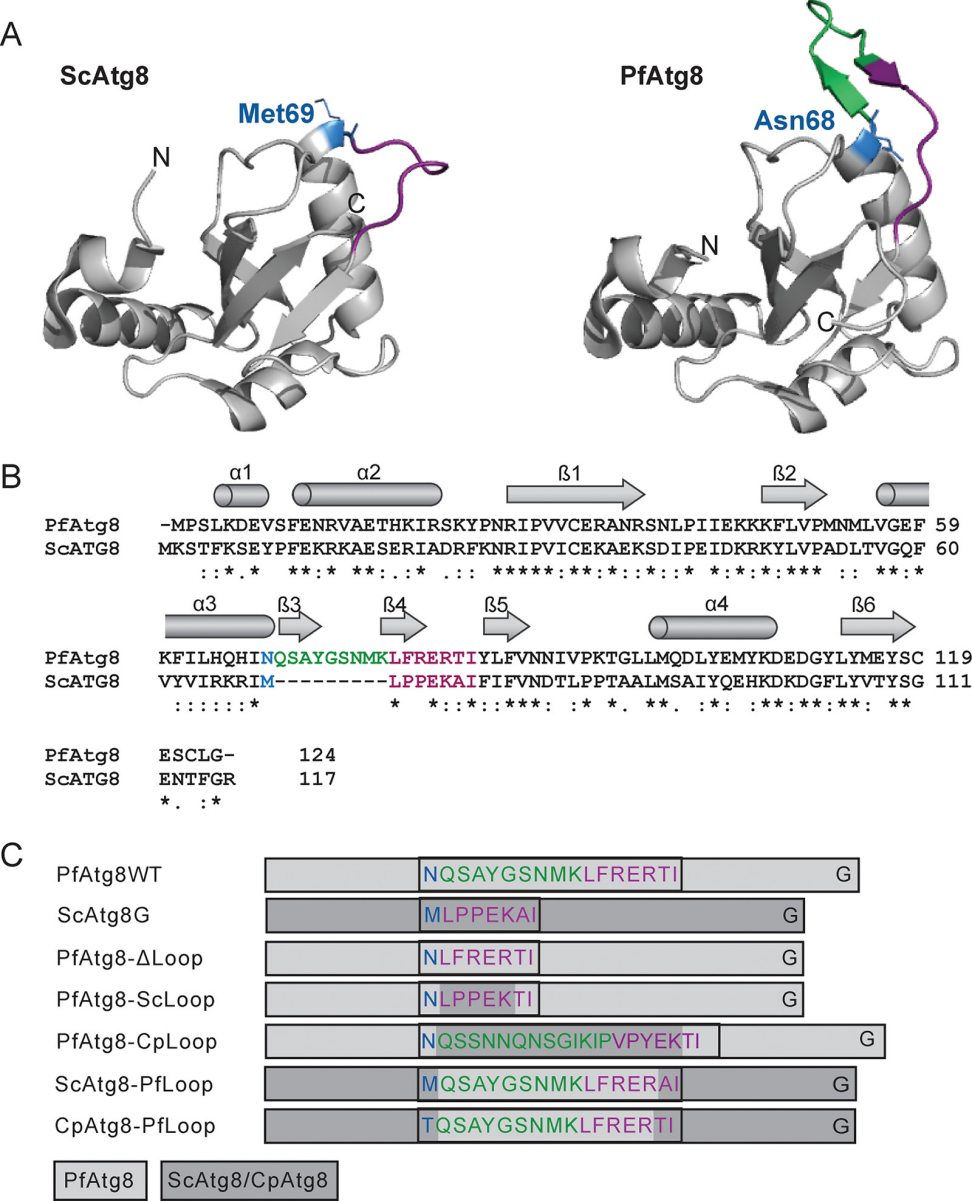
Constructs for testing the requirement for the apicomplexan-specific loop in *Pf*Atg8. (A) Structures of S. cerevisiae Atg8 (3VXW) and P. falciparum Atg8 (4EOY). The region corresponding to the loop in canonical homologs is in purple. The additional 9-amino-acid insertion in *Pf*Atg8 is in green. Amino acid residues preceding the loop region are shown as blue sticks. (B) Clustal Omega alignment of S. cerevisiae (YBL078C) and P. falciparum (PF3D7_1019900) Atg8 sequences. Color code is the same as in panel A. Secondary structure based on *Pf*Atg8 structure 4EOY (33) is indicated. *., identical amino acids; :*, similar amino acids. (C) Schematic representation of Atg8 hybrid constructs designed for testing structural requirements for Atg8 function in malaria parasites. Light gray rectangles denote *Plasmodium* sequence, and dark gray rectangles denote S. cerevisiae or C. parvum sequence. The sequence of the loop region is colored as in panel A. Note that all constructs were N terminally tagged with GFP for easier detection, which is not shown here for clarity.

The high specificity of Atg8 conjugation to the apicoplast membrane indicates that distinct structural features may govern recognition by the *Plasmodium* Atg8 conjugation machinery. Previous *in vitro* binding studies showed that interaction with *Pf*Atg3 was dependent on an extended loop region in *Pf*Atg8 (35). In the *Pf*Atg8 crystal structure, this 9-amino-acid insertion into the loop between helix α3 and strand β5 within the canonical ubiquitin fold forms a hairpin that has not previously been seen in any other homologous Atg8 structures (35) (Fig. 3A and B). Notably, Atg8 homologs and putative homologs from other apicomplexan parasites and chromerids which possess a secondary plastid also contain an insertion in this loop region, designated the apicomplexan-specific loop, though the sequence is not conserved (Fig. S2). To determine whether the extended loop motif is necessary for membrane conjugation of *Pf*Atg8, we transfected the *Pf*Atg8-TetR/DOZI strain with an episome expressing an N-terminal GFP fusion of *Pf*Atg8 in which the 9 additional amino acids (amino acids 69 to 77) were removed (*Pf*Atg8ΔLoop). In addition, since Atg8 proteins share high structural homology and because deletion constructs may be disruptive to protein folding, hybrids of *Pf*Atg8 and a canonical homolog, *Sc*Atg8, exchanging their respective loop regions, amino acids 69 to 82 in *Pf*Atg8 and 70 to 74 in *Sc*Atg8, were also constructed. Strains expressing these constructs were designated *Pf*Atg8_*Sc*Loop and *Sc*Atg8_*Pf*Loop (Fig. 3C). Finally, we constructed hybrids of *Pf*Atg8 and *Cp*Atg8 whose loop regions, *Pf*Atg8 amino acids 69 to 82 and *Cp*Atg8 amino acids 69 to 86, were exchanged (*Pf*Atg8_*Cp*Loop and *Cp*Atg8_*Pf*Loop). All constructs were N-terminally tagged with GFP.

10.1128/mbio.03642-21.2 FIG S2 Clustal alignment of the loop region and neighboring amino acids in Atg8 homologs and putative homologs from P. falciparum (*Pf*), human (*Hs*), yeast (*Sc*), T. gondii (*Tg*), C. parvum (*Cp*), other apicomplexan parasites (Neospora caninum, NCLIV_008410; Sarcocystis neurona, SN3_00202075; Hammondia hammondi, HHA_254120; Cyclospora cayetanensis, LOC34617860), and chromerids containing a secondary plastid (Vitrella brassicaformis, Vbra_15491; Chromera velia, Cvel_14496 and Cvel_25948). The additional amino acids absent from canonical homologs are in a red box. Download FIG S2, EPS file, 0.9 MB. Copyright © 2023 Walczak et al. 2023 Walczak et al. https://creativecommons.org/licenses/by/4.0/ This content is distributed under the terms of the Creative Commons Attribution 4.0 International license.

**FIG 4:**
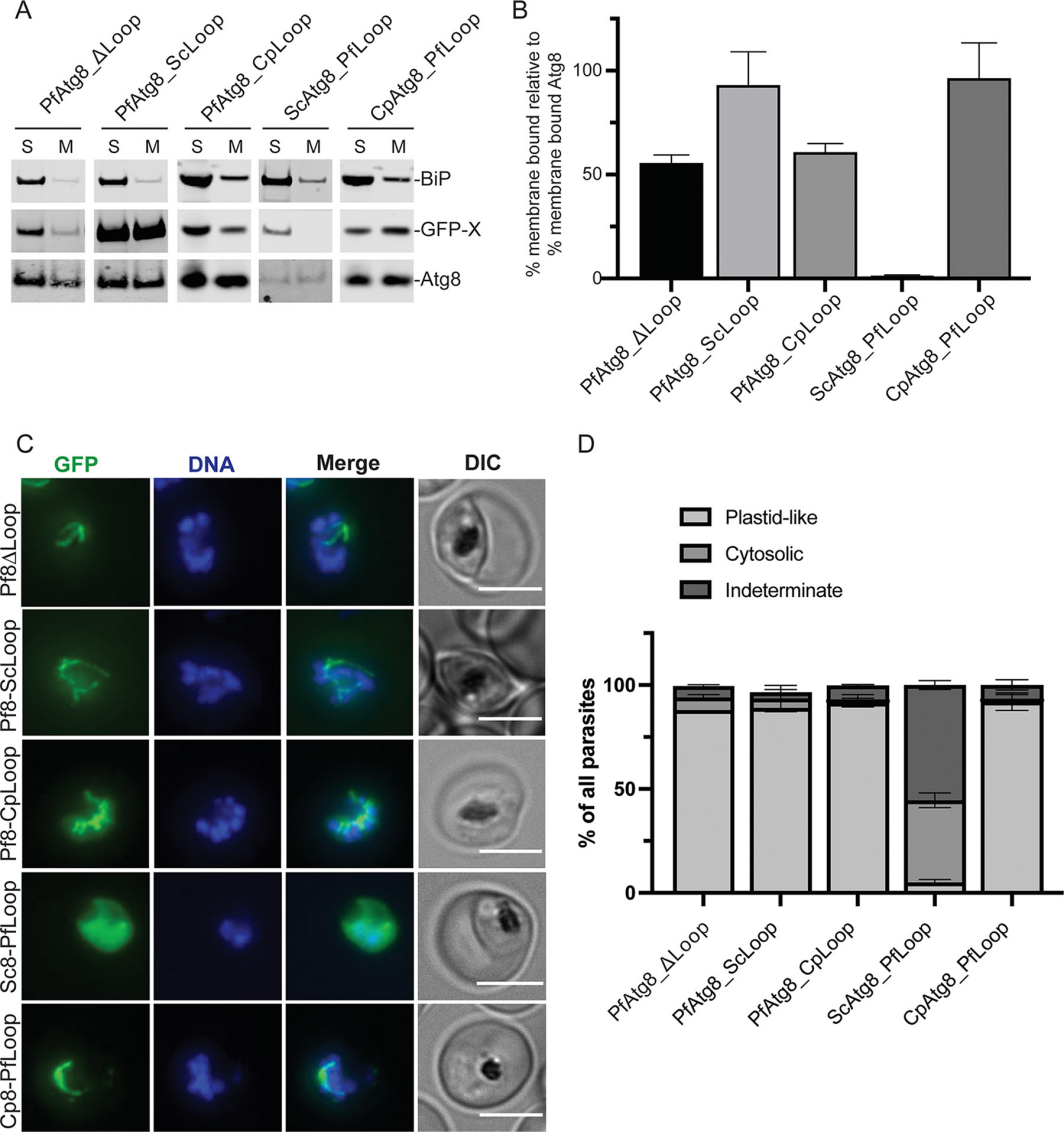
The apicomplexan-specific loop is not required for membrane conjugation of Atg8. (A) Western blot showing membrane association of indicated Atg8 mutants in a Triton X-114 fractionation experiment. S, soluble fraction; M, membrane fraction; BiP, soluble protein; Atg8, membrane-bound protein; X, Atg8 homolog. (B) Percentage of membrane-bound Atg8 mutants normalized to the percentage of the membrane-bound endogenous Atg8. Mean ± standard deviation from 2 replicates is shown. (C) Representative live microscopy images showing the localization of indicated Atg8 mutants. DNA was stained with Hoechst 33342. Maximum-intensity projections are shown. Bar, 5 μm. (D) Quantification of parasites with the indicated localization pattern of GFP-tagged Atg8 mutants. Mean ± standard deviation from 2 independent experiments is shown. For each mutant, a total of 115 to 192 cells was quantified (4 to 11 fields of view or 26 to 154 cells were recorded per each replicate).

When lysates from parasites expressing the hybrid constructs were subjected to Triton X-114 fractionation, *Pf*Atg8ΔLoop and the hybrids in which the entire *Plasmodium* loop region was replaced with the corresponding sequence from yeast or *Cryptosporidium* homologs (*Pf*Atg8_*Sc*Loop and *Pf*Atg8_*Cp*Loop) partitioned to the detergent fraction at 56%, 93%, and 61% of endogenous Atg8, respectively, indicating that these hybrids are recognized by the *Plasmodium* conjugation machinery (Fig. 4A and B). Also, *Cp*Atg8_*Pf*Loop, similar to the full-length *Cp*Atg8, partitioned to the membrane fraction comparably to *Pf*Atg8WT. In contrast, *Sc*Atg8_*Pf*Loop failed to associate with the membranes. Triton X-114 fractionation results were corroborated by live fluorescence microscopy (Fig. 4C and D). GFP-tagged *Pf*Atg8ΔLoop, *Pf*Atg8_*Sc*Loop, *Pf*Atg8_*Cp*Loop, and *Cp*Atg8_*Pf*Loop but not *Sc*Atg8_*Pf*Loop localized to structures typical of the elongated, branched, or segmented apicoplast with some cytosolic signal. *Sc*Atg8_*Pf*Loop showed cytosolic localization with occasional brighter patches that we cannot interpret.

**FIG 5:**
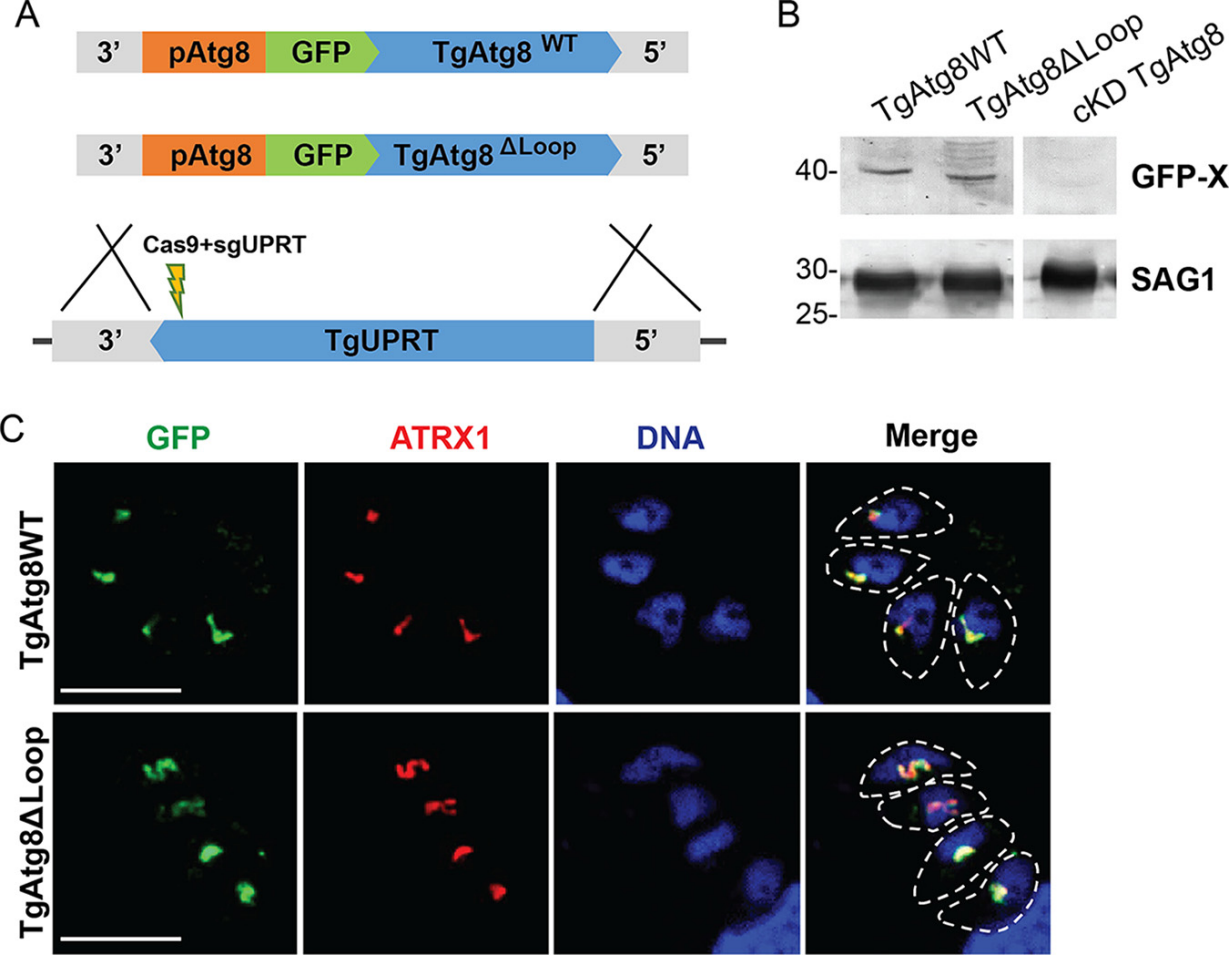
The apicomplexan-specific loop is not required for Atg8 localization to the apicoplast in T. gondii. (A) Schematic representation of introducing the GFP-*Tg*Atg8WT or *Tg*Atg8ΔLoop mutant at the uracil phosphoribosyltransferase (UPRT) locus of the cKD *Tg*Atg8 strain; pAtg8, native Atg8 promoter; 3′ and 5′ denote 3′ and 5′ untranslated regions of the UPRT gene, respectively, used as homology arms. (B) Western blot showing the expression of the GFP-fused wild-type and mutant *Tg*Atg8 as indicated; cKD *Tg*Atg8 is the parental conditional Atg8 knockdown strain. SAG1, loading control. (C) Immunofluorescence images showing colocalization of the GFP-tagged *Tg*Atg8WT or *Tg*Atg8ΔLoop with the apicoplast marker ATRX1. Parasite shape is delineated. DNA was labeled with DAPI. Bar, 5 μm.

To test whether these results hold true also for other apicomplexan species, we verified our findings in T. gondii. We constructed a GFP-tagged wild-type *Tg*Atg8 (*Tg*Atg8WT) or *Tg*Atg8ΔLoop, a mutant lacking the apicomplexan-specific loop (amino acids 68 to 76), and inserted them into the nonessential *uprt* locus of the previously described *Tg*Atg8 knockdown strain, in which Atg8 can be conditionally downregulated by the addition of anhydrotetracycline (12) (Fig. 5A). The expression of these fusion proteins, driven by the native *Tg*Atg8 promoter, was confirmed by Western blotting (Fig. 5B). When analyzed by immunofluorescence, both GFP-*Tg*Atg8WT and GFP-*Tg*Atg8ΔLoop colocalized with the apicoplast marker ATRX1 (Fig. 5C).

Taken together, and in contrast to what was previously suggested by *in vitro* binding assays (35), these results show that the apicomplexan-specific loop is not required for Atg8 recognition by the *Plasmodium* or the *Toxoplasma* conjugation machinery and for its subsequent membrane association.

### LIR-LDS interaction interface is required for apicoplast localization of *Pf*Atg8.

**FIG 6:**
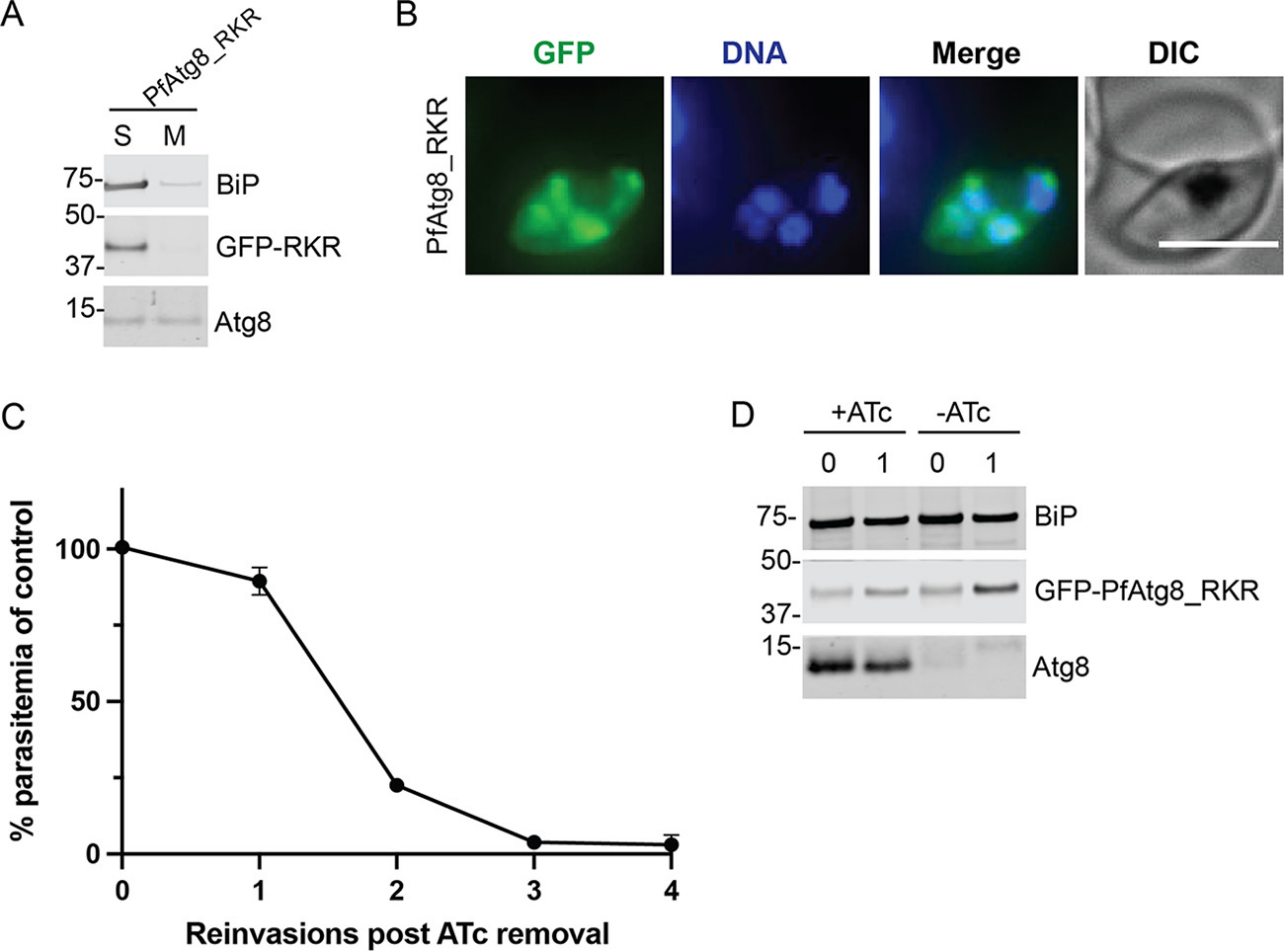
An intact LDS is required for *Pf*Atg8 membrane association and effector function. (A) Western blot showing membrane association of *Pf*Atg8_RKR mutant in a Triton X-114 fractionation experiment. S, soluble fraction; M, membrane fraction; BiP, soluble protein; Atg8, membrane-bound protein. (B) Representative live fluorescence images showing the localization of *Pf*Atg8_RKR mutant. DNA was stained with Hoechst 33342. Maximum-intensity projections are shown. Bar, 5 μm. (C) Growth of Atg8-TetR/DOZI parasites expressing GFP-*Pf*Atg8_RKR in the absence of ATc over 4 reinvasion cycles. Parasitemia for each time point was normalized to the culture grown in the presence of ATc. Mean ± standard deviation for 2 biological replicates is shown. (D) Western blots showing expression of the indicated proteins in the first two cycles of the growth time course. BiP, loading control; Atg8, endogenous Atg8. Numbers above the blot represent parasite reinvasion cycles after ATc removal.

Most known Atg8 interactions, including those with the membrane conjugation machinery and autophagic cargo adapters, occur via the LIR-LDS interface (4, 36). The LIR motif found on Atg8 effectors contains two hydrophobic amino acids separated by two random residues which bind the LDS, a surface constituted by two hydrophobic pockets on the Atg8 molecule (17, 37, 38) (Fig. S1). These interactions may be stabilized by additional contacts involving residues downstream or upstream of the LIR motif and residues outside the core LDS (3, 22, 39–41). This interaction thus involves a complex pattern of individual residues in the Atg8 sequence. Notably, a patch of positively charged amino acids, RKR or RRR, at the end of the α3 helix in mammalian LC3 and GABARAP homologs has been implicated in binding to residues adjacent to LIR motifs and modulating the selectivity and/or affinity of the LIR-LDS interactions (22, 42, 43). The corresponding region of *Pf*Atg8 (residues 66 to 68) is likely less basic as it consists of amino acids HQH (Fig. S1). We wondered whether this change was important for LIR-LDS interactions with the conjugation machinery or perhaps a new effector interaction. Therefore, we tested a *Pf*Atg8 mutant in which the residues HQH were replaced with RKR (*Pf*Atg8_RKR) for apicoplast membrane association and functional complementation. In the *Pf*Atg8 crystal structure, H66 of *Pf*Atg8 forms a hydrogen bond with the LIR motif of Atg3, in addition to the typical interaction within the hydrophobic pockets of the core LDS (35). Consistent with an interaction with membrane conjugation enzymes, *Pf*Atg8_RKR did not show membrane binding in a Triton X-114 fractionation assay, did not localize to the apicoplast, and consistently failed to restore growth of parasites lacking Atg8 (Fig. 6A to D). These results confirm that, at a minimum, the LIR-LDS interface is required for the apicoplast localization of *Pf*Atg8. Because mutation of the LDS disrupted *Pf*Atg8 apicoplast localization, we were unable to assess its contribution to downstream effector recruitment. This region may also be required for other reported autophagic functions of *Pf*Atg8 that were not assessed (23).

### The apicomplexan-specific loop does not determine the apicoplast-specific function of Atg8.

**FIG 7:**
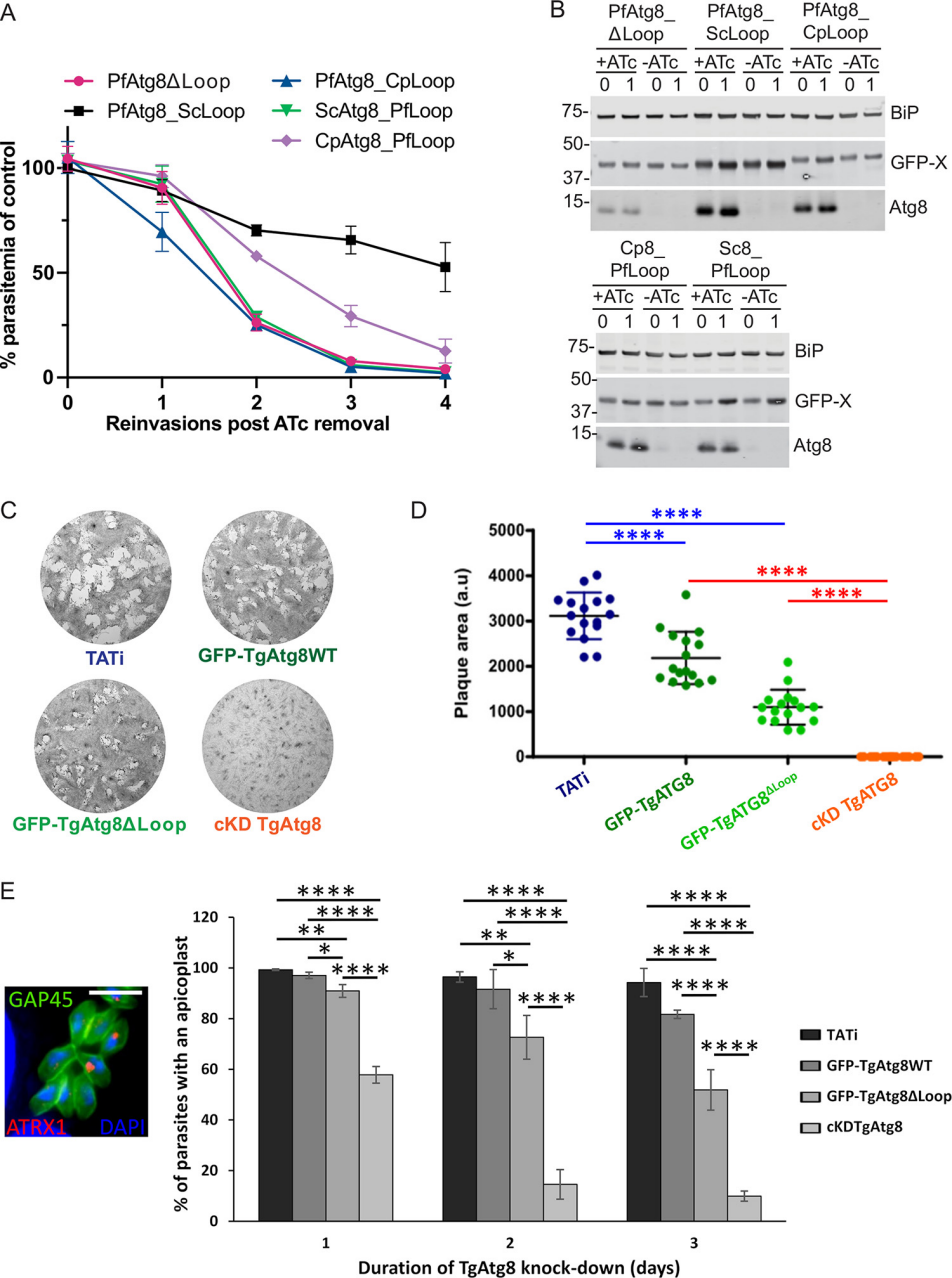
The effector function of apicomplexan Atg8 requires a region outside the apicomplexan-specific loop. (A) Growth of Atg8-TetR/DOZI *Plasmodium* parasites expressing indicated GFP-tagged Atg8 mutants in the absence of ATc over 4 reinvasion cycles. Parasitemia for each time point was normalized to the culture grown in the presence of ATc. Mean ± standard deviation for 2 biological replicates is shown. (B) Western blots showing expression of the indicated proteins in the first two cycles of the growth time course. BiP, loading control; Atg8, endogenous Atg8; X, Atg8 mutant. Numbers above the blot represent parasite reinvasion cycles after ATc removal. (C) Plaque assay performed for 8 days in the continuous presence of ATc to deplete the regulatable copy of the endogenous *Tg*Atg8. The TATi parental cell line was used as a control. (D) Mean plaque area ± standard deviation for one representative experiment out of *n* = 3 as shown in in panel C. a.u., arbitrary unit. Statistical significance was evaluated by multiple comparisons of groups with one-way analysis of variance followed by a Tukey *post hoc* test. ****, *P* < 0.0001. (E) Quantification of apicoplast loss by immunofluorescence microscopy after depleting regulatable *Tg*Atg8 for the indicated number of days. (Left inset) Example of image used for quantification; parasite shape is labeled with anti-GAP45, DNA is labeled with DAPI, and the apicoplast is labeled with anti-ATRX1. Bar, 5 μm. The TATi parental cell line was used as a control. Represented are mean values ± standard deviations from *n* = 3 experiments. Counting was performed on at least 200 cells (200 to 310 individual cells, depending on the data set). Statistical significance was evaluated by multiple comparisons with one-way analysis of variance followed by a Tukey *post hoc* test. *, *P* < 0.05; **, *P* < 0.01; ****, *P* < 0.0001.

Notably, *Cp*Atg8 is conjugated to the apicoplast membrane (indicating an intact LDS-LIR interaction) but is unable to rescue growth defects in parasites lacking endogenous Atg8 (Fig. 1C and D and Fig. 2A and B). This result suggested that *Pf*Atg8 effector function requires regions outside the LDS. To date, very few effectors have been shown to interact with Atg8 proteins using alternative interaction sites (19, 20). We wondered if the apicomplexan-specific loop might be involved in a new interaction for *Pf*Atg8 effector function at the apicoplast. The Atg8 loop hybrids that were able to conjugate to the apicoplast membrane (see above) were further tested for their ability to rescue growth defects in cells lacking endogenous Atg8. While *Pf*Atg8ΔLoop, *Pf*Atg8_*Cp*Loop, and *Cp*Atg8_*Pf*Loop could not rescue growth inhibition in Atg8-deficient parasites, parasites expressing *Pf*Atg8_*Sc*Loop could replicate in the absence of endogenous Atg8, albeit at a lower rate than in the presence of endogenous Atg8 (Fig. 7A and B).

We also assessed the apicomplexan-specific loop requirement for replication of T. gondii. Lytic growth of intracellular *Toxoplasma* parasites over a period of days results in formation of plaques within monolayers of host cells. The plaque size positively correlates with the ability of the parasites to replicate and invade new host cells. Complementation with *Tg*Atg8ΔLoop partially restored parasite growth and apicoplast inheritance upon depletion of the endogenous Atg8, although less efficiently than *Tg*Atg8WT (Fig. 7C to E).

In conclusion, the extended apicomplexan-specific loop is not strictly required for the apicoplast-specific function of either *Plasmodium* or *Toxoplasma* Atg8. The apicomplexan-specific loop may instead have a more general role, for example, in maintaining protein stability. Thus, in *Plasmodium* and, by analogy, in *Toxoplasma*, too, another region outside the LDS is likely required for the effector function.

### Investigating the function of previously identified Atg8 effector regions in *Pf*Atg8 function.

**FIG 8:**
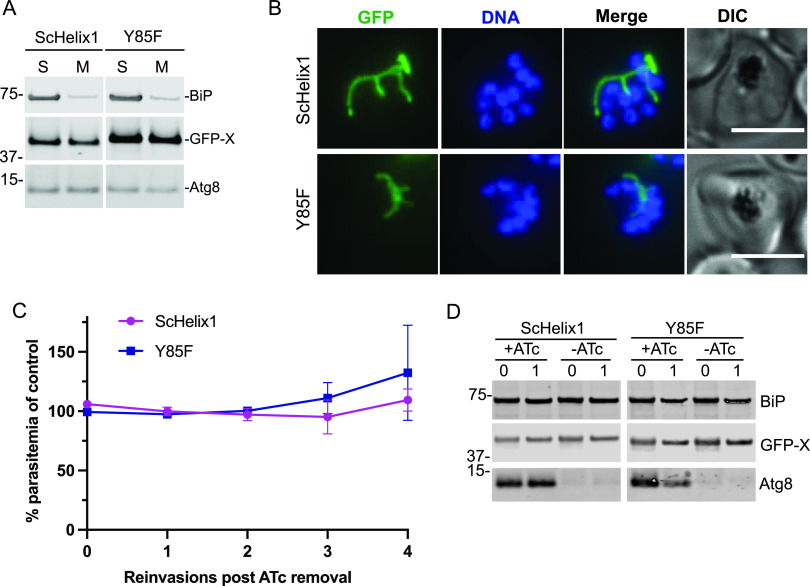
Previously identified interaction sites on Atg8 are not required for *Pf*Atg8 apicoplast function. (A) Western blot showing membrane association of indicated *Pf*Atg8 mutant in a Triton X-114 fractionation experiment. S, soluble fraction; M, membrane fraction; BiP, soluble protein; Atg8, membrane-bound protein; X, Atg8 homolog. (B) Representative live fluorescence images showing the localization of the indicated *Pf*Atg8 mutants. DNA was stained with Hoechst 33342. Maximum-intensity projections are shown. Bar, 5 μm. (C) Growth of Atg8-TetR/DOZI parasites expressing indicated GFP-tagged *Pf*Atg8 mutants in the absence of ATc over 4 reinvasion cycles. Parasitemia for each time point was normalized to the culture grown in the presence of ATc. Mean ± standard deviation for 2 biological replicates is shown. (D) Western blots showing expression of the indicated proteins in the first two cycles of the growth time course. BiP, loading control; Atg8, endogenous Atg8. Numbers above the blot represent parasite reinvasion cycles after ATc removal.

In addition to the LDS, other regions of Atg8 have been implicated in intermolecular interactions of canonical Atg8 homologs. The N-terminal helical region, which generally has little sequence conservation between homologs, has been implicated in oligomerization, binding of tubulin and lipids, and membrane tethering and fusion. It is also thought to contribute to the selectivity of LIR-LDS interactions (2, 18, 19, 44). To test whether this region is required for *Pf*Atg8 function, we replaced the α1 N-terminal helix of *Pf*Atg8 (amino acids 1 to 9) (Fig. S1) with the corresponding region from yeast Atg8 (amino acids 1 to 10) and transfected it into Atg8-TetR/DOZI parasites. This construct, named *Sc*Helix1, correctly localized to the apicoplast, was membrane bound, and rescued parasite growth upon depletion of endogenous Atg8 (Fig. 8A to D). We also attempted to replace both N-terminal helices with the corresponding region of yeast Atg8 but never obtained transfectants. Altogether, our data show that the most N-terminal helix is not specifically required for Atg8 function in the *Plasmodium* apicoplast.

A recently identified UDS (Fig. S1) serves as a binding site for proteins containing a UIM, including a number of autophagy receptors and the Atg4 protease (20, 45, 46). Its consensus sequence is Ψ-F-Ψ-Ω/T, where Ψ is hydrophobic and Ω is aromatic. The second residue, Phe, is highly conserved in most canonical homologs, is critical for interactions with the effectors, and plays a role in induction of membrane budding *in vitro* and regulation of vacuolar morphology *in vivo* (20, 47). *Pf*Atg8, as well as its homologs or putative homologs from apicomplexans and chromerids, has a tyrosine residue in this position (Tyr85). We therefore replaced Tyr85 with Phe and episomally expressed it as a GFP fusion in Atg8-TetR/DOZI parasites. This mutant, designated Y85F, also bound membranes, showed apicoplast localization, and restored growth of parasites lacking endogenous Atg8 (Fig. 8A to D). In contrast, a *Tg*Atg8 mutant where the tyrosine was mutated to an alanine was recently described and shown to be affected in its localization and function at the apicoplast but not in its lipidation (48). Overall, this suggests that while the UDS may be important for Atg8 function at the apicoplast, the central tyrosine residue found in Apicomplexa species can be replaced by the canonical phenylalanine residue found in mammalian, yeast, or plant Atg8 sequences.

## DISCUSSION

Atg8 family proteins are critical players in the cell’s endomembrane system with diverse functions in autophagy and nonautophagic pathways (3, 49). Although they are part of one of the most highly conserved eukaryote-specific protein families, little is known about the molecular basis for Atg8 functional diversification, particularly for nonautophagic functions. In *Plasmodium* and related Apicomplexa members, the single Atg8 ortholog has evolved an essential function in the biogenesis of the apicoplast, a unique plastid of secondary endosymbiosis origin (11, 16). Here, we used a functional complementation approach to determine structural requirements for this unusual function of apicomplexan Atg8. We showed that the new effector function of apicomplexan Atg8 requires interactions other than those previously identified for macroautophagy and selective autophagy functions. The LDS, important for interaction with the membrane conjugation machinery, was shown to be important for apicoplast localization of *Pf*Atg8, and the overall importance of this docking site was also recently demonstrated for *Tg*Atg8 (48). On the other hand, mutation of the N-terminal helix did not affect the apicoplast-related function. Surprisingly, even the apicomplexan-specific loop was not required for membrane binding or cellular effector function. Atg8 proteins and their conjugation machinery are highly conserved among eukaryotes, and it is presumed that a pathway involving Atg8 was present in the “last eukaryotic common ancestor” (LECA) (5). The secondary endosymbiosis origin of the apicoplast indicates that the apicoplast function was a later adaptation of Atg8 proteins. However, an intriguing question remains: was autophagy or another membrane-related pathway the ancestral function of Atg8? The pathways in which Atg8 family proteins function ultimately involve vesicular transport of material between compartments of the endomembrane system, including the endoplasmic reticulum (ER), Golgi apparatus, plasma membrane, and the vacuole/lysosome (50–57). However, the functional diversification of Atg8 proteins beyond autophagy is most apparent in organisms that possess multiple Atg8 orthologs, like mammals or plants. Instead, most eukaryotes, from fungi, protists, and sponges to higher multicellular organisms, encode at least a minimal set of Atg proteins and have a version of autophagy or an autophagy-like pathway manifested by the formation of double-membrane vesicles called autophagosomes (15, 58–62). Moreover, autophagosome formation and subsequent canonical degradative autophagy are induced by nutrient limitation in an almost universal way in a wide variety of eukaryotes. Based on this evolutionary pattern, autophagy was most likely the ancestral function of Atg8 and subsequent environmental adaptations led to diversification of Atg8 function through expansion, reduction, or complete loss of autophagy machinery (15, 63). Studies of Atg8 in a greater diversity of organisms will provide more support for the evolutionarily ancestral function of Atg8.

If we assume autophagy was the ancestral function of Atg8 proteins, it is intriguing how such a compact protein (<150 amino acids) proceeded to evolve so many diverse nonautophagic functions. Atg8 proteins generally function as membrane-bound scaffolds that recruit other factors. We hypothesize that *Pf*Atg8 acts in a similar manner, i.e., by recruiting other proteins that in turn carry out the effector functions. Most known Atg8 interactions occur via the LDS on Atg8, which binds LIR motifs of the other proteins (4). Additionally, the N-terminal helical region which distinguishes Atg8 proteins from other ubiquitin-like proteins has also been implicated in Atg8 interactions (19, 20). Our work shows that while the LDS seems to be important for the apicoplast-related role for Atg8 in Apicomplexa, the N-terminal region is not, which highlights specificities in the regulation of Atg8 function in these eukaryotes. Studies in mammalian Atg8 isoforms have shown that their functional specialization can be due to relatively subtle sequence differences, which can lead to different receptor-binding properties (37, 41). Hence, we looked for specificities in the apicomplexan Atg8 sequences.

The most obvious candidate was the loop between the helix α3 and the β-strand β5 in *Pf*Atg8 structure. This loop is longer in *Pf*Atg8 and its homologs from apicomplexans and chromerids than in canonical Atg8 proteins. Surprisingly, this extended loop is also not strictly required for the effector function because when replaced with the corresponding (shorter) sequence from yeast, such an Atg8 mutant can still partly rescue parasites lacking endogenous Atg8. Its replacement with the *Cryptosporidium* loop or deletion of the additional 9 amino acids in the *Plasmodium* loop renders the protein nonfunctional. Is it required for another function like stabilizing the protein structure? And most importantly, what are the structural features responsible for the effector function of *Pf*Atg8? Recent findings on the targeting of *Tg*Atg8 (48) suggest that it is translocated to the apicoplast via vesicles in a SNARE-dependent manner, suggesting that the protein may be directly conjugated to the outermost membrane of the apicoplast but to vesicular intermediates first: it was described that the mutation of the UDS abolished *Tg*Atg8 localization on the apicoplast but not its lipidation (potentially to the trafficking vesicles). This illustrates a potential additional layer of complexity in the apicoplast-specific function of Atg8 and apicomplexan-specific features that are likely driven by sequence and structural determinants.

Structural data are needed to answer these questions and better understand the structure-function relationship for the unique role of Atg8 in *Plasmodium*. We attempted to crystallize *Toxoplasma* and *Cryptosporidium* Atg8 homologs as well as the loop mutants of *Pf*Atg8; unfortunately, our attempts were so far unsuccessful. Identifying effectors that interact with *Pf*Atg8 on the apicoplast membrane will also provide insights into the molecular mechanism of Atg8 in apicoplast biogenesis. Strains generated in this study, expressing Atg8 mutants that correctly localize to the apicoplast membrane but have lost the effector function, could be used in a comparative coimmunoprecipitation/mass spectrometry (co-IP/MS) analysis to identify specific Atg8 interaction partners required for its role in apicoplast biogenesis.

## MATERIALS AND METHODS

### Cloning of *Plasmodium* constructs.

Primers and gBlocks used in this study are listed in Table S1 in the supplemental material. All constructs for genetic complementation were cloned into pfYC110 using In-Fusion (TaKaRa Bio). Atg8 homologs were cloned without the C-terminal extension following the last glycine residue. To clone pfYC110-GFP-*Pf*Atg8WT, GFP with a Gly-Ala-Gly-Ala linker and AatII site was amplified from pfYC110-ACP_L_-GFP, and *Pf*Atg8 was PCR amplified from *Plasmodium* genomic DNA. The two fragments were inserted into pfYC110, resulting in plasmid pfYC110-GFP-*Pf*Atg8WT. *Pf*Atg8 was removed from that plasmid by AatII-SacII digest and replaced with Atg8 homologs. Human Atg8 homologs LC3A, LC3B, GABARAP, and GABARAPL1 were amplified from cDNA libraries kindly provided by the labs of S. Pfeffer and L. Li; gBlocks (IDT DNA) were used for cloning human LC3C and GABARAPL2; *Sc*Atg8G was amplified from yeast S288C genomic DNA. To clone the *Plasmodium*-yeast loop hybrids, parts of Atg8 from the start codon to the loop or from the loop to the stop codon were amplified from the plasmids carrying *Pf*Atg8 or *Sc*Atg8 and the desired loop sequence was added on the primer; the N- and C-terminal parts of the hybrid were then fused by overlap extension PCR; the N-terminal GFP tag was amplified with a Gly-Ala-Gly-Ala linker and PvuI site downstream of it; both fragments were simultaneously inserted into pfYC110 via AvrII-SacII restriction sites. The remaining hybrid constructs as well as CpAtg8 were purchased as gBlocks (IDT DNA) and inserted into pfYC110 via AvrII-SacII sites. Accession numbers for the aligned sequences are as follows: hLC3A isoform 1, NP_115903; hLC3B, NP_073729; hLC3C, NP_001004343; hGABARAP, NP_009209; hGABARAPL1 isoform 1, NP_113600; hGABARAPL2, NP_009216; *Pf*Atg8, PF3D7_1019900; *Tg*Atg8, TGME49_254120; *Cp*Atg8, CPATCC_0009830.

10.1128/mbio.03642-21.3 TABLE S1 Primers and gBlocks used in this study. Download Table S1, XLSX file, 0.01 MB. Copyright © 2023 Walczak et al. 2023 Walczak et al. https://creativecommons.org/licenses/by/4.0/ This content is distributed under the terms of the Creative Commons Attribution 4.0 International license.

### *Plasmodium* culture and transfection.

Plasmodium falciparum parasites were grown in human erythrocytes (Stanford Blood Center, Stanford, CA) at 2% hematocrit under 5% O_2_ and 5% CO_2_, at 37°C in RPMI 1640 medium supplemented with 5 g/L AlbuMAX II (Gibco), 2 g/L NaHCO_3_ (Fisher), 25 mM HEPES (pH 7.4) (Sigma), 0.1 mM hypoxanthine (Sigma), and 50 mg/ L gentamicin (Gold Biotechnology) (further referred to as culture medium). For transfections, 200 μL packed red blood cells per transfection was washed twice in Cytomix, combined with 50 to 100 μg ethanol-precipitated plasmid DNA dissolved in 30 μL Tris-EDTA (TE) buffer and to 170 μL Cytomix and transferred to a 0.2-cm electroporation cuvette. Erythrocytes were electroporated at 310 V, 950 μF, and infinite resistance using a Gene Pulser XCell electroporation system (Bio-Rad). Electroporated red blood cells were added to schizont-stage parasites from 0.5 mL culture at 5% parasitemia, 2% hematocrit. For drug selection, the following drugs were used as applicable starting 3 days after transfection: 2.5 mg/L blasticidin S (RPI Research Products), 2.5 nM WR99210, and 500 μg/mL G418 sulfate (Corning); 0.5 μM anhydrotetracycline (Sigma) was added to parasites to maintain expression of TetR/DOZI-regulated genes.

### Generation of complemented *Tg*Atg8 cell lines.

Tachyzoites of the T. gondii cKD *Tg*Atg8 cell line (12), as well as derived transgenic parasites generated in this study, were maintained by serial passage in a human foreskin fibroblast (HFF; American Type Culture Collection; CRL 1634) cell monolayer grown in Dulbecco’s modified Eagle’s medium (DMEM; Gibco), supplemented with 5% decomplemented fetal bovine serum, 2-mM l-glutamine, and a cocktail of penicillin-streptomycin at 100 μg/mL.

Complemented cell lines were generated by insertion of an additional *Tg*Atg8 copy at the uracil phosphoribosyltransferase (UPRT) locus in the cKD *Tg*Atg8 mutant (12). The pGFP-*Tg*Atg8 plasmid (64) was used as a template with primers ML2463 and ML2464, and the products were self-ligated to generate p-GFP-TgAtg8Δ68-76 excluding amino acids 68 to 76 (QCAQNSGLP). A 1.5-kbp sequence corresponding to the promoter region of *Tg*Atg8 was obtained by PCR from genomic DNA with primers ML2429 and ML2430 and cloned with NsiI upstream of the GFP-*Tg*Atg8 or GFP-*Tg*Atg8ΔLoop fragment in its respective plasmid. These were then used as a PCR template to amplify, with primers ML2624 and ML2625, a cassette containing the *Tg*Atg8 promoter followed by the sequence coding for GFP-fused wild-type (WT) or truncated *Tg*Atg8. These cassettes were cloned using NotI and XmaI into the pUPRT-TUB-Ty plasmid (65) to yield the pUPRT-GFP-*Tg*Atg8 and pUPRT-GFP-*Tg*Atg8ΔLoop plasmids, respectively. These plasmids were then linearized with KpnI and BamHI prior to transfection into the cKD *Tg*Atg8 cell line (12) together with a plasmid expressing Cas9 and a UPRT-specific guide RNA under the control of a U6 promoter (66). Then transgenic parasites were selected with 5 μM fluorodeoxyuridine and cloned by limiting dilution. Primers are listed in Table S1.

### *Plasmodium* growth assays.

Ring-stage parasites at 5% to 10% parasitemia were washed twice in the culture medium to remove ATc and resuspended in the culture medium, and the hematocrit was adjusted to 2%. Parasites were divided into 2 cultures grown in the culture medium supplemented with 0.5 μM ATc or grown in the medium without ATc. The growth assays were continued for 4 reinvasion cycles. At the schizont stage of each cycle, parasitemia was measured by flow cytometry starting at 24 h (0 reinvasions) after ATc removal. Culture aliquots were incubated with 17 μM dihydroethidium (Thermo Fisher) for 15 min at room temperature (RT) to stain nuclei, and 50,000 events were analyzed on a BD Accuri C6 flow cytometer. Cultures were diluted with fresh culture medium with red blood cells at 2% hematocrit so that the parasitemia under the +ATc condition was 1% and the −ATc condition was diluted by the same factor; ATc was added as required. Aliquots of culture for Western blotting were taken at 24 h and 72 h (0 and 1 reinvasion) after ATc removal.

### Plaque assays.

Confluent monolayers of HFFs were infected with freshly egressed parasites, which were left to grow for 7 days in the absence or presence of 1 μg/mL anhydrotetracycline (ATc). They were then fixed with 4% (vol/vol) paraformaldehyde, and plaques were revealed by staining with a 0.1% crystal violet solution (catalog no. V5265; Sigma-Aldrich).

### Western blotting.

For *Plasmodium*, erythrocytes were lysed with 0.1% saponin for 5 min on ice to release parasites. Parasite pellets were washed twice with ice-cold phosphate-buffered saline (PBS), resuspended in 1× lithium dodecyl sulfate buffer (Life Technologies) in PBS supplemented with 25 μM dithiothreitol (DTT), and boiled for 5 min at 96°C. After separation on Bis-Tris Novex gels (Invitrogen), proteins were transferred to a nitrocellulose membrane and blocked with a blocking buffer containing 0.1% Hammarsten-grade casein (Affymetrix), 0.2× PBS, and 0.01% sodium azide. Next, membranes were incubated with primary antibodies diluted in a wash buffer consisting of 50% blocking buffer, 50% Tris-buffered saline, and 0.25% Tween (TBST) overnight at 4°C or 1 h at room temperature, followed by 3 washes with TBST and 1 h of incubation with the secondary antibodies diluted in the same buffer. Blots were visualized using the LiCor double-color detection system. Blot images were converted to grayscale images for the purpose of this publication and were analyzed using Image Studio Lite software. Primary antibodies and dilutions used in this study were as follows: guinea pig anti-*Pf*Atg8 serum (Josman LLC), 1:1,000; mouse anti-GFP (Clontech; 632381), 1:10,000; mouse anti-Plasmodium yoelii BiP (a gift from Sebastian Mikolajczak and Stefan Kappe), 1:20,000. Secondary fluorophore-conjugated antibodies were purchased from Fisher (LiCor) and used at a 1:10,000 dilution.

For T. gondii, protein extracts from 10^7^ freshly egressed tachyzoites were prepared in Laemmli sample buffer, separated by SDS-PAGE, and transferred onto nitrocellulose membrane using the Bio-Rad Mini Trans-Blot system according to the manufacturer’s instructions. Mouse anti-GFP monoclonal antibody (catalog no. 11814460001; Roche; clones 7.1 and 13.1) was used to detect tagged proteins, and mouse anti-SAG1 monoclonal antibody (67) was used as a loading control.

### Triton X-114 fractionation assay.

Schizont-stage parasites were lysed with 0.1% saponin and washed 3 times with ice-cold PBS. Parasite pellets were resuspended in ice-cold lysis buffer (1× PBS, 1% Triton X-114 [Thermo Scientific 28332], 2 mM EDTA, 1× protease inhibitors [Pierce A32955]) and incubated on ice for 30 min. Cell debris was removed by a 10-minute centrifugation at 16,000 × *g*, 4°C. Supernatant was transferred to a fresh Eppendorf tube, incubated for 2 min at 37°C to allow phase separation, and centrifuged for 5 min at 16,000 × *g* at room temperature. The top (aqueous) layer was transferred to another tube. The interphase was removed to avoid cross-contamination between the layers. The bottom (detergent) layer was resuspended in 1× PBS-0.2 mM EDTA to equalize the volumes of the two fractions. Both fractions were subjected to methanol-chloroform precipitation, resuspended in PBS containing 2× NuPAGE lithium dodecyl sulfate sample buffer, boiled for 5 min at 95°C, and analyzed by Western blotting. Quantification of band intensities was done using Image Studio Lite. The percentage of each GFP fusion protein that was membrane bound was calculated and normalized to the percentage of endogenous Atg8 in the membrane.

### Live microscopy.

Live P. falciparum parasites in PBS with 0.4% glucose were incubated with 2 μg/mL Hoechst 33342 stain (Thermo Fisher H3570) for 15 min at room temperature to visualize nuclei. Images were acquired using the Olympus IX70 microscope equipped with a DeltaVision Core system, a 100× 1.4-numerical-aperture (NA) Olympus lens, a Sedat Quad filter set (Semrock), and a CoolSnap HQ charge-coupled device (CCD) camera (Photometrics) controlled via SoftWoRx 4.1.0 software. Images were acquired as z-stacks and analyzed using ImageJ. Unless stated otherwise, maximum-intensity projections are shown in the figures.

### Immunofluorescence microscopy.

For immunofluorescence assays (IFAs) on T. gondii, intracellular tachyzoites grown on coverslips containing HFF monolayers were fixed for 20 min with 4% (wt/vol) paraformaldehyde (PFA) in PBS, and permeabilized for 10 min with 0.3% Triton X-100 in PBS. Coverslips were subsequently blocked with 0.1% (wt/vol) bovine serum albumin (BSA) in PBS. Primary antibodies used were mouse monoclonal anti-ATRX1 (68) and rabbit anti-GAP45 (69). Staining of DNA was performed on fixed cells by incubating them for 5 min in a 1-μg/mL 4′,6-diamidino-2-phenylindole (DAPI) solution. Images were acquired at the Montpellier RIO imaging facility from a Zeiss Axio Imager Z1 epifluorescence microscope driven by the ZEN software v2.3 (Zeiss).
